# Application of intentional facial nerve stimulation during cochlear implantation as an electrophysiological tool to estimate the intracochlear electrode position

**DOI:** 10.1038/s41598-022-17732-9

**Published:** 2022-08-04

**Authors:** David P. Herrmann, Franz-Tassilo Müller-Graff, Stefan Kaulitz, Mario Cebulla, Anja Kurz, Rudolf Hagen, Tilmann Neun, Kristen Rak

**Affiliations:** 1 Department of Oto-Rhino-Laryngology, Plastic, Aesthetic and Reconstructive Head and Neck Surgery and the Comprehensive Hearing Center, University of Wuerzburg, Josef-Schneider-Str. 11, 97080 Wuerzburg, Germany; 2 Department of Diagnostic and Interventional Neuroradiology, University of Wuerzburg, Josef-Schneider-Str. 11, 97080 Wuerzburg, Germany

**Keywords:** Neuroscience, Medical research, Neurology, Engineering

## Abstract

This proof of concept describes the use of evoked electromyographic (EMG) activation of the facial nerve for intraoperative monitoring of the electrode insertion during cochlear implantation (CI). Intraoperative EMG measurements from the facial nerve were conducted in nine patients undergoing CI implantation. Electric current pulses were emitted from contacts on the CI array during and immediately after electrode insertion. For control, the results of EMG measurements were compared to postoperative flat panel volume computed tomography scans with secondary reconstruction (fpVCT_SECO_). During insertion, the EMG response evoked by the electrical stimulation from the CI was growing with the stimulating contact approaching the facial nerve and declined with increasing distance. After full insertion, contacts on the apical half of the CI array stimulated higher EMG responses compared with those on the basal half. Comparison with postoperative imaging demonstrated that electrode contacts stimulating high EMG responses had the shortest distances to the facial nerve. It could be demonstrated that electrically evoked EMG activation of the facial nerve can be used to monitor the progress during CI electrode insertion and to control the intracochlear electrode position after full insertion.

## Introduction

Cochlear implantation (CI) has developed as the most effective rehabilitation method for patients with high degrees of hearing loss over the last decades^1^. Presupposition for a good speech understanding is the correct position of the electrode in the cochlea. Thus, an unfavorable intracochlear placement of the electrode like dislocation or a tip fold over has to be avoided, since this will lead to an inappropriate stimulation of the neuronal structures in the cochlea^2,3^. In a recent review it was described, that incorrect electrode position, like incomplete insertion or kinking occurs more often in straight flexible electrodes, while electrode tip-fold over can more often be detected in pre-formed electrodes^4^. On the other hand the correct positioning of the electrode in combination with an anatomically based selection of electrode stimulation can lead to a better speech understanding, since an optimized cochlear coverage can be obtained^5–10^. To control the position of the electrode, different techniques are available either real-time during insertion or after the insertion process. Real-time control is possible using intraoperative imaging. The only technique, which can directly detect electrode position, is the fluoroscopy^11–13^. Disadvantages of this technique are that the scan has to be performed in the Stenver´s projection and the ionizing radiation exposure at least to the surgeon and the patient. In addition, real-time monitoring of the electrode insertion process is possible by electrocochleography (ECochG). During ECochG recording of signals elicited via hair cells and auditory nerve fibers the relative position of the electrode can be measured^14^. Increasing signal amplitudes in intracochlear ECochG recordings could be observed during CI electrode insertion^15^. However, as ECochG recordings depend on responses of residual hearing function of the cochlear^14^, patients can show different patterns of signal behavior^16^ impeding the usage of ECochG as a tool to locate the CI array’s position.

Different tools have been developed to intraoperatively detect electrode dislocation or malposition either using electrode-neuronal interactions or systems, which use the electric activity of the implant. By the measurement of electrically induced compound action potentials the scalar location^17^ and a tip fold over^18^ can be detected. In addition, the impedances^19^ or the spread of excitations^20^ can be used to detect an unfavorable electrode position. After the insertion or after the operation radiological imaging is normally used to examine the position of the electrode. Various systems are available, which mostly use ionizing radiation to control the electrode position^3,10,11^, but there is ongoing research to use magnetic resonance imaging (MRI) for control after cochlear implantation^21^.

Facial nerve monitoring (FNM) is widely used in neurosurgical^22^, neurotological^23,24^ and other head and neck interventions, such as parotid surgery^25^. By FNM, the electromyographic activity (EMG) of the muscles innervated by the facial nerve is monitored to measure the function of the seventh cranial nerve indirectly. In CI, FNM is widely used to avoid unintended nerve injury during drilling in the mastoid and the posterior tympanotomy^26^, but in some studies it was shown, that the application of FNM does not reduce the risk of harming the facial nerve^27^. FNM is also used for navigation in robotic cochlear implantation^28^.

CIs can in principle stimulate the facial nerve electrically. This effect is the basis of postoperative facial nerve stimulation (FNS), a rare but unwanted side effect of cochlear implantation. Typical symptoms caused by FNS, are involuntary twitching of facial muscles or even painful spasm of facial muscles^29^. Postoperative FNS may be facilitated by a number of known conditions, such as otosclerosis^30,31^, cochlear malformations^32^ or in cases with a significantly reduced thickness of bone separating the upper basal turn of the cochlea and the labyrinthine segment of the facial nerve^33,34^.

The results of a computational model by Frijns et al.^35^, suggested that otosclerosis can cause the stimulation threshold of the facial nerve to lie within the electrical dynamic range of the CI user. FNS occurring post implantation can be reduced by using triphasic pulse stimulation^36,37^, electrode repositioning, change of implant type or finally by explantation^38^. On the other hand, FNS can theoretically be triggered in any CI user if sufficiently high stimulation levels are applied^36^. However, as these stimulation levels are above the maximum comfortable loudness level in CI users who do not exhibit the previously described conditions, they can only be applied in anesthetized subjects.

Since all the previously mentioned measures to control for the intracochlear positioning of the electrode have disadvantages there is still a demand for additional tools to provide the surgeon with real-time feedback during the implantation procedure. For this purpose, the aim of the present study was to develop a new electrophysiological technique. In this regard, FNS, which is normally regarded as an unwanted side effect of CI, was intentionally evoked during surgery and evaluated for its applicability to estimating the electrode position.

The basic hypothesis is that the EMG signal induced by the stimulation of electrode contacts in close proximity to the facial nerve is different to distant electrode contacts. Thus, the different signals can possibly be used to estimate the relative distance of the respective electrode contact to the facial nerve. To test this, a concept was developed in which the most apical electrode contact was used for FNS during insertion. Hypothetically, the EMG signal will rise in real-time, when the electrode contact approximates to the facial nerve in its labyrinthine portion. After passing the facial nerve and during increase of the distance the EMG signal should decline. By this procedure a pass of the electrode contact at the area of the facial nerve can be measured, which is a sign of a relative progress of insertion. After full insertion, stimulation is performed from every electrode contact. Hypothetically, the EMG signal should be highest for the electrode contact closest to the facial nerve. Thus, a map can be generated, which shows the distance between every contact and the facial nerve, which could be used to measure full insertion.

## Methods

### Subjects

The study was performed in 9 adult patients (Hereafter referred to as subjects and abbreviated as S1–S9), who underwent cochlear implantation at the Department of Oto-Rhino-Laryngology, Plastic, Aesthetic and Reconstructive Head and Neck Surgery at the University of Wuerzburg. Seven women and 2 men were included, ranging in age from 29 to 84 (mean = 53.67, SD = 22.72) years (see Table 1 for demographic details). All subjects fulfilled the indication for cochlear implantation according to the German Sk2 guideline for cochlear implantation and were implanted with a SNYCHRONY2® cochlear implant system from the manufacturer MEDEL GmbH (Innsbruck, Austria). This manufacturer offers a range of electrode types with different array lengths to provide an optimal fit of the implant to the patient's anatomy. The participating subjects were implanted with 4 different electrode types (see Table 1). Table 2 lists the electrode types used, their respective array length, and the spacing between the individual contacts. In the present study, the electrode contacts on the arrays were numbered from apical to basal in ascending order.

**Table 1:** Demographic details of the subjects. Subjects are numbered according to the order in which they participated in the study.

Subject	Age	Sex	Electrode type	Etiology
S1	34	F	Standard	Otosclerosis
S2	29	F	FLEX28	Neurofibromatosis type II
S3	84	F	FLEX28	Idiopathic
S4	71	M	FLEX28	Idiopathic
S5	57	F	Standard	Idiopathic
S6	58	F	FLEX28	Sudden sensorineural hearing loss
S7	76	F	FLEXsoft	Sudden sensorineural hearing loss
S8	32	M	FLEX24	Idiopathic
S9	32	F	FLEX24	Idiopathic

**Table 2:** Details of the different electrode types included.

Electrode type	Electrode array length [mm]	Active stimulation length [mm]	Intercontact spacing [mm]
Standard	31.5	26.4	2.4
FLEXsoft	31.5	26.4	2.4
FLEX28	28.0	23.1	2.1
FLEX24	24.0	20.9	1.9

The study was performed following the declaration of Helsinki and approved of the Ethics Committee of the University of Wuerzburg (315/15_z). Prior to surgery, all subjects were informed about the study and gave their written informed consent.

### Experimental setup

**Figure 1:**
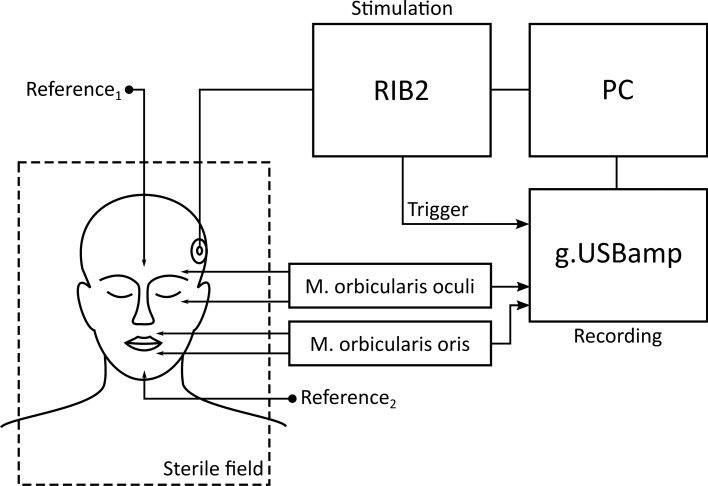
Experimental setup for intraoperative EMG recordings. The setup consisted of a PC for stimulation and measurements control, a research CI interface (RIB2) for stimulation and a g.USBamp biosignal amplifier to record two EMG channels.

The experimental setup (see Figure 1) was based on the systems introduced by Bahmer et al.^36^. It consisted of a research interface box 2 (RIB2; Department of Ion Physics and Applied Physics at the University of Innsbruck, Austria) and a g.USBamp biosignal amplifier (g.tec GmbH, Schiedlberg, Austria) connected to a laptop computer (2.5 GHz Dual Core Intel CPU and 8 GB RAM). All stimulation and recording parameters were controlled via a graphical user interface programmed with the software Matlab R2017b (The MathWorks Inc., Natick, USA). The RIB2 converted the stimulation parameters into commands that were sent to the subject’s CI via a telemetric transmitter coil. The biosignal amplifier recorded the EMG response to the stimulation pulses via subcutaneous needle electrodes, which were inserted ipsilaterally into two different groups of muscles innervated by the facial nerve. The EMG channel recording the EMG activity of the M. orbicularis oculi measured the potential differences between an electrode above the eyebrow and one below the eye. The electrodes of the channel recording the EMG activity of the M. orbicularis oris were inserted above the upper lip and below the lower lip. The reference electrodes were placed in inactive areas on the forehead and the chin. It was verified that all impedances were below 5 kΩ. To synchronize stimulation and EMG recordings, the RIB2 emitted a trigger signal on the onset of each pulse. The trigger signal was fed to an input of the biosignal amplifier. The inputs of the biosignal amplifier were sampled at a rate of 38.4 kHz. The digitized signals were stored on the hard drive of the computer.

### Stimulation parameters

The stimulation consisted of biphasic pulses with cathodic-leading phase applied in monopolar mode. The phase duration of each pulse was set to 150 µs and the interphase gap to 2.1 µs. For each measurement, 2 subsequent pulses were emitted with a rate of 100 pulses per second.

### Real-time measurements

**Figure 2:**
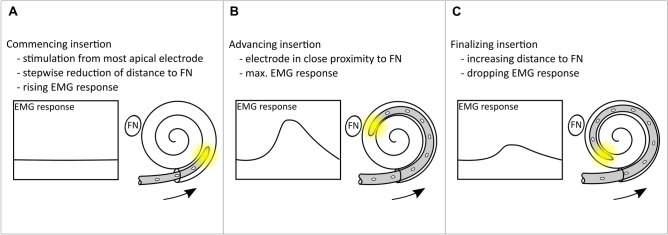
Schematic illustration of the real-time measurements. EMG activity is recorded during the stepwise insertion of the cochlear implant electrode array. Panels (**A**, **B**, and **C**) illustrate three different stages of the measurements procedure. The facial nerve is indicated by the abbreviation FN.

The procedure to measure the electrode insertion in real-time included the following steps: First, the surgeon inserted two electrode contacts into the tympanic scale and paused while the test conductor started the stimulation from the most apical contact with a low current level. If the applied current level did not evoke an EMG response, the current level was carefully increased until an EMG response was recorded that was just visually discernable from the background noise of the biosignal amplifier. The following step included the surgeon continuing the insertion by the next two electrode contacts and then pausing. During the pause, stimulation was performed with the current level determined in the first step and the corresponding EMG response was measured. This step was repeated for two electrode contact at a time until full electrode insertion was achieved. Figure 2 describes three theoretical key stages of the measurement procedure during stepwise insertion. In Figure 2A, only a small number of electrode contacts are inserted into the cochlea. The distance between the most apical contact and the facial nerve is still far, thus stimulation evokes only minor EMG responses. In Figure 2B, the insertion has already advanced. Because of the immediate proximity, the most apical contact can stimulate the facial nerve most effectively and evoke a high EMG response. In Figure 2C, the most apical contact has already passed the facial nerve and the distance between the two is increasing again. As a result, the stimulation loses its effectiveness and the EMG response decreases with each insertion step.

### Post insertion measurements

After full insertion of the electrode, stimulation was performed from every electrode contact on the array and the subsequent EMG response was recorded from both EMG channels. For each subject, multiple measurement runs with different stimulation levels were conducted to gain data with optimal signal-to-noise ratio. The current level used for the real-time measurements was selected as the start value. If this current level also produced clear EMG responses from the individual electrode contacts with the electrode fully inserted, lower current levels were used in subsequent measurement runs. However, if this current level did not elicit any detectable EMG responses, the current level was increased until a detectable response was measured.

### Radiological measurements

**Figure 3:**
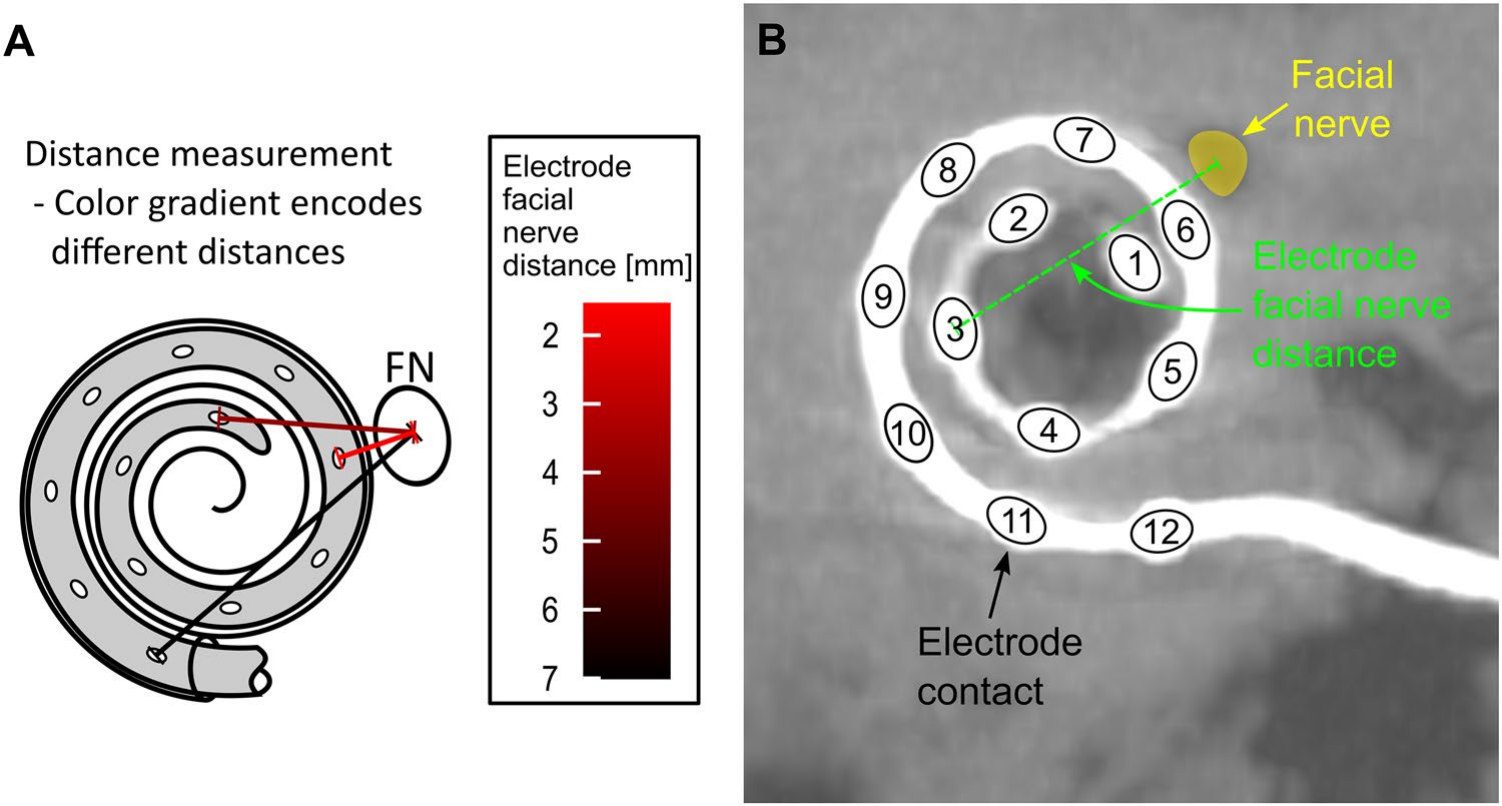
(**A**) Schematic illustration of the measurement of the electrode facial nerve distance with color-coding of the relative distances (red: shortest distance; black: longest distance). (**B**) Example of the measurement of the electrode facial nerve distance in the cochlear view of an fpVCT_SECO_ scan. The electrode contacts of the CI are numbered in order from apical to basal. The facial nerve canal is marked in yellow in the scan. The dashed green line symbolizes the distance between the center of the facial nerve and the electrode contact number 3.

For control of the insertion flat panel volume computed tomography (fpVCT) scans were performed on an angiographic system (Axiom Artis; Siemens Healthcare AG, Erlangen, Germany) with commercially available software Syngo DynaCT (Siemens Healthcare AG) resulting in a slice thickness of 466 µm. Subsequently, secondary reconstructions (fpVCT_SECO_) with a slice thickness of 99 µm were created^39^. All CT scans were converted to the DICOM format and imported to the open-source medical image viewer Horos (Version 3.3.5; Nimble Co LLC d/b/a Purview, Annapolis, Maryland USA). First, the “cochlear view”^40^ was set, which is an axial view of the cochlea generated by rotation against the three body axes. In this two-dimensional view, the electrode facial nerve distance (EFND) for each electrode was measured between the center of the metal artifact and the center of the facial nerve canal in cross section. Figure 3A is a schematic illustration of the EFND measurement. Figure 3B is an example of an fpVCT_SECO_ scan in cochlear view depicting the CI electrode contacts with their position on the array, the facial nerve, and the EFND of electrode contact number 3.

### Statistics

The data were analyzed with the programming language for statistical computing R (version 4.0.2) and the RStudio (version 1.3.1073) integrated developments environment. The ggplot2 package (3.3.5) was used for graphical representation of the data. In order to investigate the correlation between EFNDs and normalized EMG amplitudes, the nonparametric Spearman’s rank correlation was calculated using the stats package (4.0.2).

## Results

### Measurement of the electrode facial nerve distance

**Figure 4:**
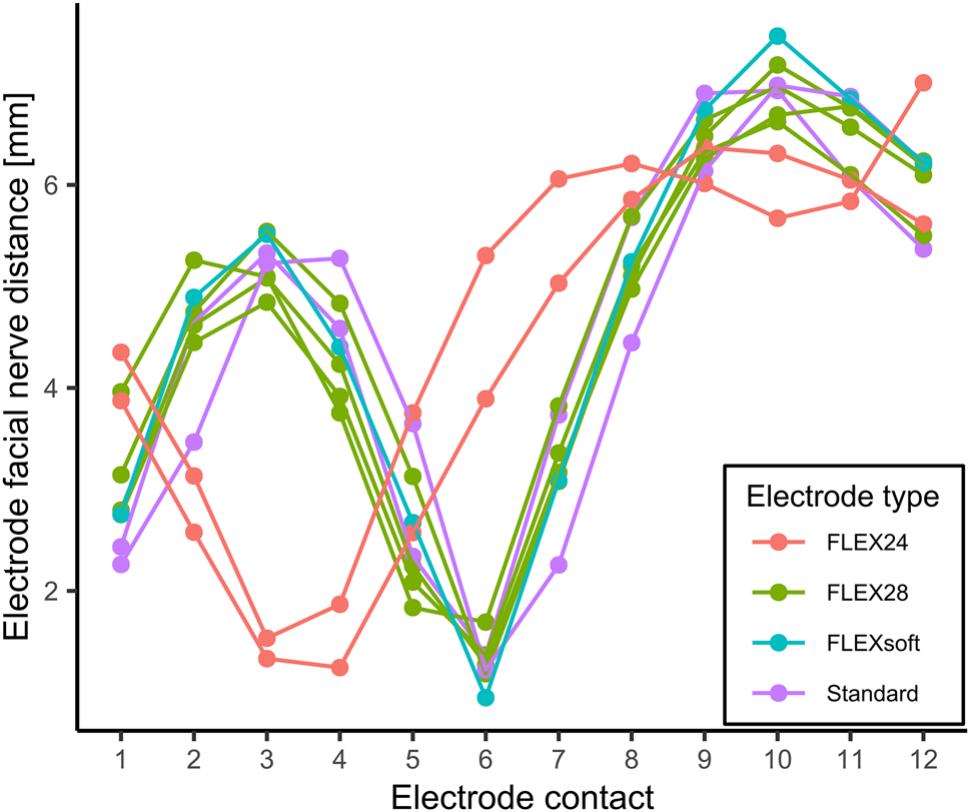
Electrode facial nerve distance as function of the electrode contact number. Distances were measured from fpVCT_SECO_ scans in cochlear view. Colors correspond to different implanted electrode types.

As can be seen in Figure 4, fpVCT_SECO_ of subjects implanted with the electrode type Standard, FLEXsoft, or FLEX28 showed the shortest EFND for the medial electrode contact number 6. The shortest EFND of S8 and S9, both implanted with the short electrode type FLEX24, was measured for electrode contact number 3 and 4, respectively.

### Real-time insertion of the electrode

During insertion, a subject-specific current level was used to stimulate the EMG response. The current level used for the respective subject can be found in the second column of Table 3. The third column of Table 3 contains the corresponding charge level in charge units (QU), with 1 QU approximately corresponding to 1 nC.

**Table 3:** Stimulation levels used for evoking EMG responses during both measurement procedures.

Subject	Current level used during electrode insertion [CU]	Charge level used during electrode insertion [QU]	Current level used for the post insertion measurement [CU]	Charge level used for the post insertion measurement [QU]
S1	614.25	92.138	519.753	77.963
S2	–	–	1134	170.1
S3	378	56.7	378	56.7
S4	472.5	70.875	661.5	99.225
S5	472.5	70.875	311.85	46.778
S6	472.5	70.875	519.75	77.963
S7	378	56.7	378	56.7
S8	425.25	63.788	425.25	63.788
S9	378	56.7	330.75	49.613

Column 2 and 4 contain the current levels used for the real-time measurement and the post insertion measurement, respectively. Column 3 and 5 contain the corresponding charge levels for columns 2 and 4.

In 8 of the 9 subjects, an EMG response was continuously recorded on both channels during the stepwise insertion of the electrode. S2 was excluded from the analysis because no EMG response could be evoked until a number of 6 contacts had been inserted and thus no conclusion could be drawn about half of the insertion process. In subject S1, recording began when 4 contacts had already been inserted instead of two. The charge levels used during insertion ranged between a minimum of 56.7 QU and a maximum of 92.138 QU with a mean value of 67.331 QU.

**Figure 5:**
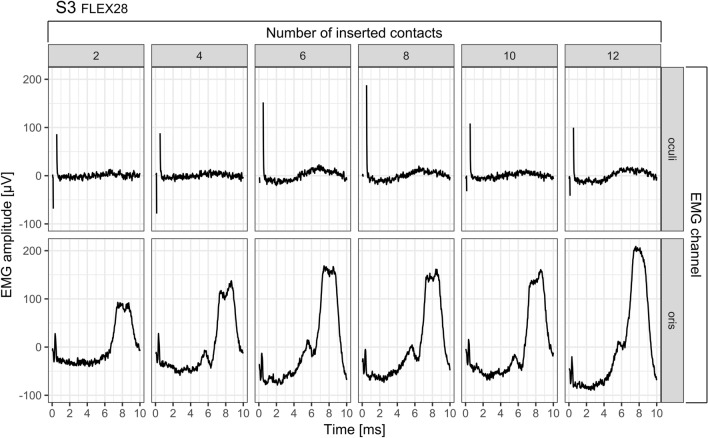
Example of a raw EMG recording during electrode insertion in subject S3. For each insertion step, the stimulation current level was 378 CU. The upper row shows the EMG recordings of M. orbicularis oculi, the lower row of M. orbicularis oris. The number of currently inserted contacts can be read from the number line above the graph. Each signal corresponds to the average of the EMG responses to two subsequently emitted stimulation pulses. In both EMG channels, the electrical stimulation artifact caused by the stimulation pulse can be seen at the beginning of each recording. In the case of the orbicularis oculi muscle, the artifact is several times the amplitude of the artifact of the orbicularis muscle and is therefore clipped in this representation.

As an example, Figure 5 depicts the raw EMG recordings during the stepwise insertion process in subject S3. The upper and the lower row show the EMG curves for the periocular and perioral EMG channel respectively. The number of the currently inserted channels is indicated by the number above each graph. The curves shown are the mean value of the EMG signals evoked by the two subsequent stimulation pulses. The electrical stimulation artifact caused by the stimulation pulse can be seen at the beginning of each recording. In the case of the periocular EMG channel, the artifact is several times the amplitude of the artifact of the perioral EMG channel and is clipped because of the chosen y-axis scaling. The comparison of the amplitude of the EMG responses between the two channels clearly shows that the response strength recorded with the perioral channel was a multiple of the amplitude in the periocular channel. In the course of the stepwise insertion in subject S3, it can be seen how the EMG amplitudes in both channels increase with each step until a number of 6 electrode contacts have been inserted. As the insertion of 8 and 10 electrode contacts progresses, the EMG response decreases again, but increases a second time at complete insertion of 12 electrode contacts. In the case of the periocular canal, the EMG response reaches its maximum at 6 inserted electrode contacts; in the case of the perioral canal, the maximum is reached only at complete insertion.

EMG response strength differed greatly between subjects as well as between the periocular and perioral EMG channels. Thus, to achieve better comparability of the results, the data of each subject and each EMG channel were normalized. The normalization process assigned the value 0 to the lowest EMG response and 1 to the maximum EMG response.

**Figure 6:**
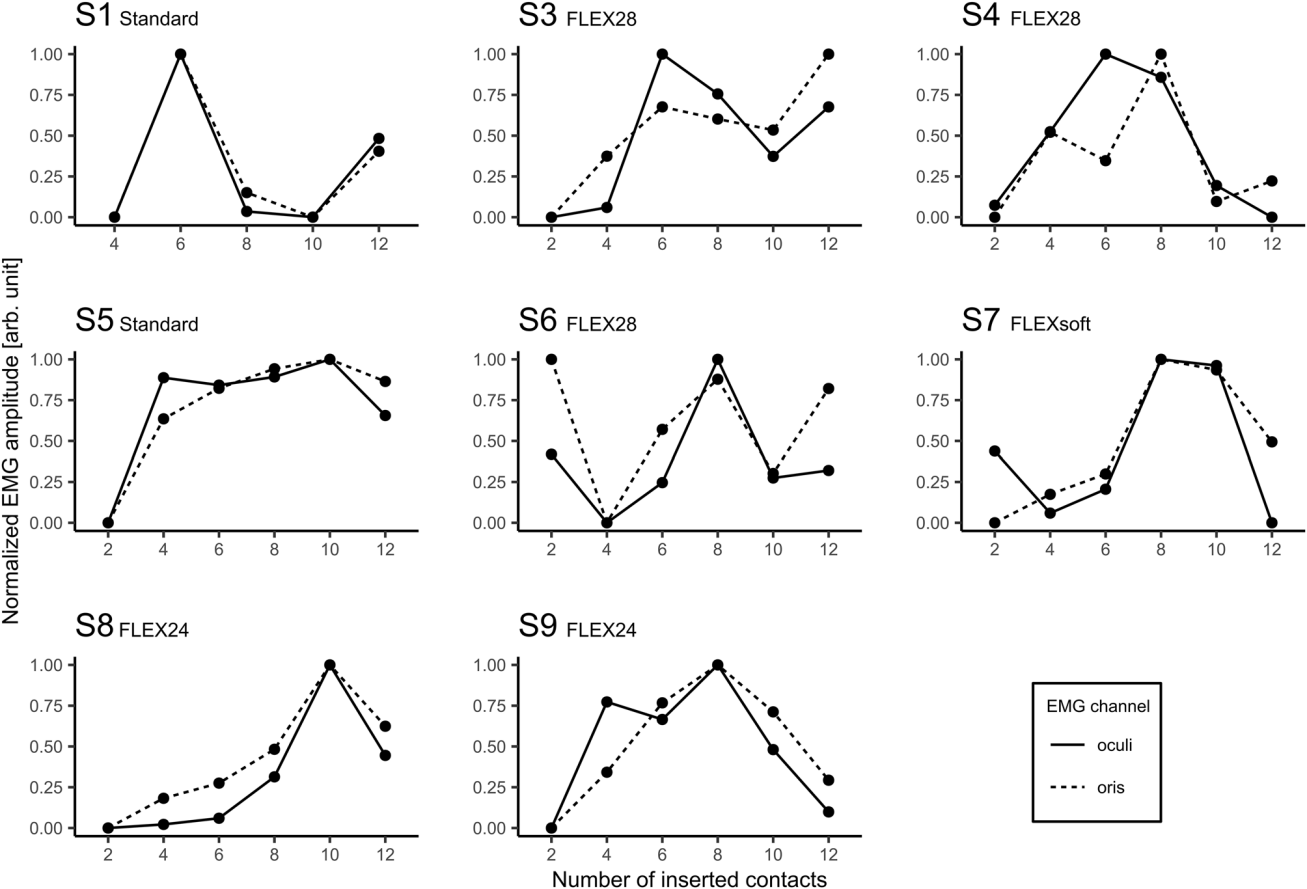
Normalized EMG responses during the stepwise electrode insertion in 8 subjects. The EMG responses are shown as function of the number of inserted electrode contacts. The solid and dashed lines correspond to the periocular and perioral EMG channel, respectively. Normalization assigned the value 0 to the lowest amplitude and 1 to the maximum amplitude.

Figure 6 shows the normalized EMG response during the stepwise insertion for both EMG channels. With few exceptions, the measurements of both EMG channels demonstrated a stable concordance for all of the 8 subjects, an initial increase and subsequent decrease in the normalized EMG response can be seen as the insertion progressed.

Figure 6 shows that in the cases of S1, S3, and S6 the EMG response increases again towards the end, that is, when the array was fully inserted. The measurements in S4 demonstrated peaks of the EMG signal at two different numbers of inserted contacts (periocular: 6; perioral: 8). For the remaining subjects, the peaks appearing at the medial portion of the cochlea revealed a stable concordance between both EMG channels. Under the condition of an exactly constant step size during insertion, it can be assumed that the position P of the stimulating apical contact can be calculated as the difference between the number of all contacts on the array (all MED-EL electrode types feature 12 contacts) and the number of already inserted contacts N_inserted_. During the stepwise insertion, the EMG amplitude could be continuously tracked in 8 out of 9 subjects.

1 $$P=12-{N}_{inserted}$$

Taking into account the deflection of the EMG signal in respect to P, the contact that is closest to the facial nerve after full insertion can now be estimated. Table 4 lists the values of P calculated by formula 1 which show the highest EMG response in each channel and compares them with the respective shortest measured EFND. The comparison revealed a clear concordance between imaging and EMG measurement in 5 of 7 (EMG periocular) and 4 of 7 (EMG perioral) subjects. With respect to S8, contact 3 shows the shortest EFND. Since step sizes of 2 contacts were used during insertion, an exact agreement cannot be determined for odd contact numbers. However, at S8 the peak at the neighboring contact 2 is visible.

**Table 4:** Comparison between the shortest radiologically measured EFND per subject and the estimated position of the contact showing a clear peak in both EMG channels during insertion.

Subject	Shortest EFND	P_oculi_	P_oris_
S1	6	6	6
S3	6	6	6
S4	6	6	4
S5	6	2	2
S6	6	6	6
S7	6	4	4
S8	3	2	2
S9	4	4	4

### Post insertion measurements

**Figure 7:**
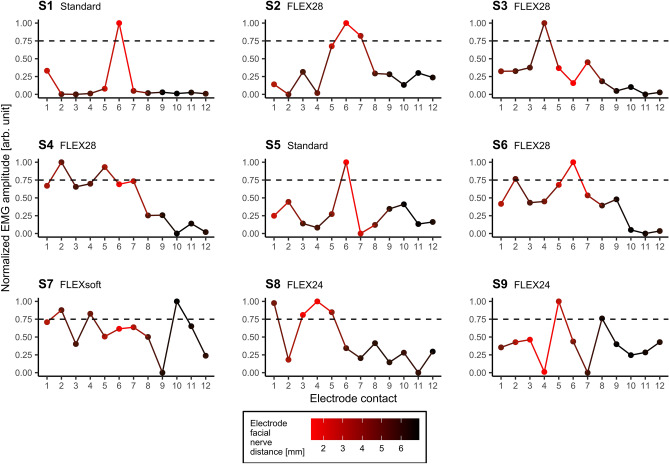
Normalized EMG responses of the M. orbicularis oculi as function of the contacts on the fully inserted electrode array. The color gradient depicts the radiologically determined electrode facial nerve distance (red: shortest distance; black: longest distance) for each subject. The horizontal dashed line indicates the arbitrarily chosen EMG threshold at 75% normalized EMG response.

**Figure 8:**
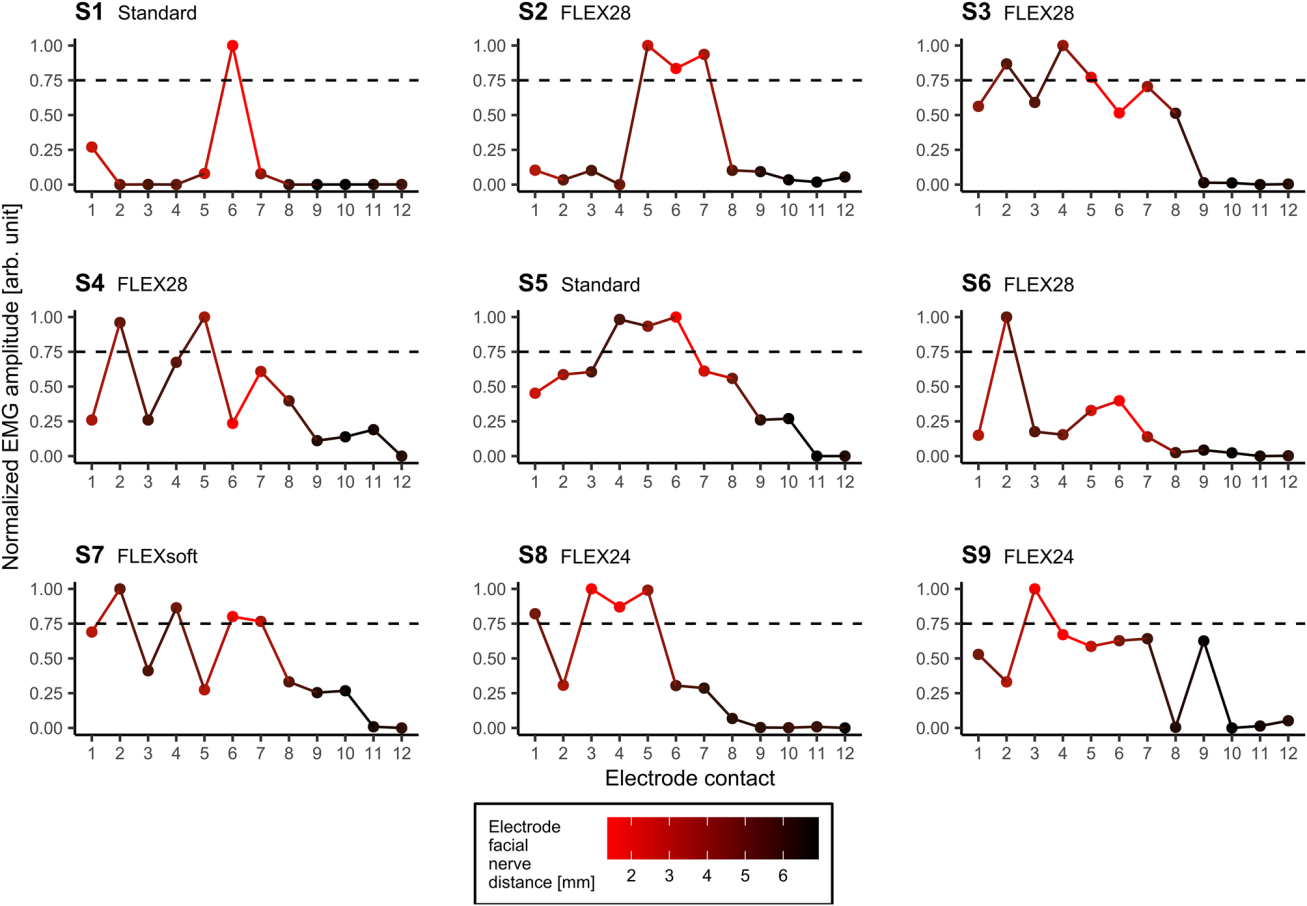
Normalized EMG responses of the M. orbicularis oris as function of the contacts on the fully inserted electrode array. The color gradient depicts the radiologically determined electrode facial nerve distance (red: shortest distance; black: longest distance) for each subject. The horizontal dashed line indicates the arbitrarily chosen EMG threshold at 75% normalized EMG response.

The current levels used for the post insertion measurements varied from the current levels used during the real-time measurements. Column 4 and 5 of Table 3 show the subject-specific current levels and the corresponding charge levels, respectively. Each subject showed an individual stimulation threshold, above which a visually clearly detectable EMG response could be observed. The charge levels used during insertion ranged between a minimum of 46.778 QU and a maximum of 170.1 QU with a mean value of 77.648 QU. However, too high stimulation levels could result in no clear peaks depending on the electrode contact used. Such a “breakdown” consequently caused strong EMG responses on several contacts of the array, which prevent estimations of the position of the electrode with respect to the facial nerve by means of the EMG amplitudes. Therefore, only those data were included in the analysis, which were recorded using stimulation amplitudes that elicited a clear EMG response and allowed the best possible spatial differentiation. EMG responses of the M. orbicularis oculi are depicted in Figure 7, those of the M. orbicularis oris in Figure 8 after stimulation from every contact of the fully inserted electrode array for each of the 9 subjects. As an additional dimension, the radiologically measured EFNDs per contact of the respective subject were plotted as a relative color gradient to the curves in Figures 7 and 8. As for the real-time measurements, the EMG response of each subject and each EMG channel was normalized for better comparability. Normalization assigned the value 0 to the lowest EMG response and 1 to the highest. As evaluation criterion for the presence of a spike in EMG data, a threshold at 75% normalized EMG response was arbitrarily defined. As can be seen in Figures 7 and 8, the selected current levels on single and multiple electrode contacts, respectively, achieved response strengths that exceeded 75% normalized EMG amplitude. The EMG responses that exceeded the threshold are subsequently referred to as peaks. Table 5 summarizes at which electrode contact the shortest EFND was measured radiologically, and at which contacts EMG peaks were measured after complete insertion.

**Table 5:** Listing of electrode contacts per subject with the shortest electrode facial nerve distance (EFND) and EMG peaks in the periocular and perioral EMG channels, which exceeded the threshold at 75% normalized EMG amplitude.

Subject	Shortest EFND [electrode contact]	Peaks periocular [electrode contact]	Peaks perioral [electrode contact]
S1	6	6	6
S2	6	6, 7	5, 6, 7
S3	6	4	2, 4, 5
S4	6	2, 5	2, 5
S5	6	6	4, 5, 6
S6	6	6	2
S7	6	2, 4, 10	2, 4, 6, 7
S8	3	1, 3, 4, 5	1, 3, 4, 5
S9	4	5	3

**Figure 9:**
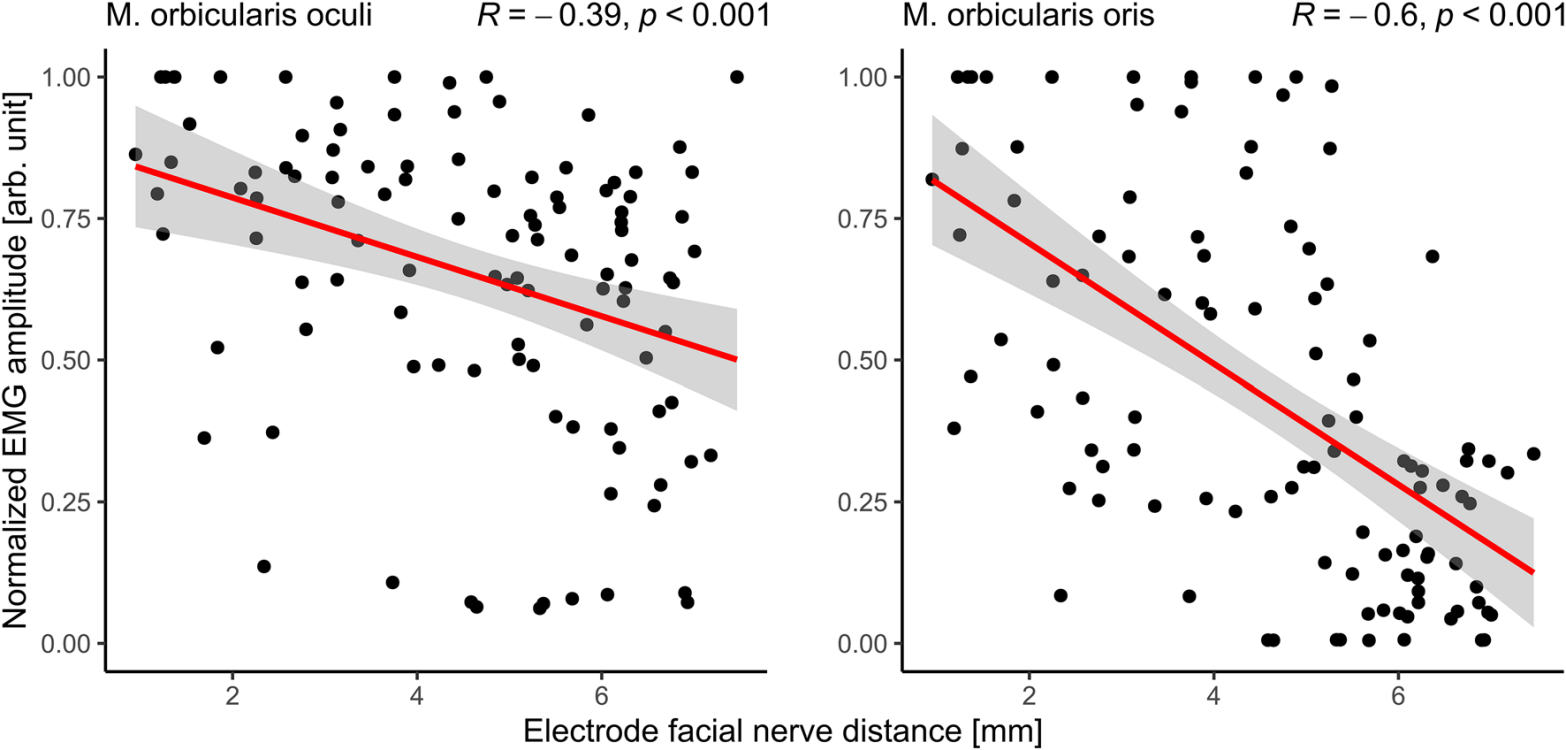
Correlation between the normalized EMG responses of the M. orbicularis oculi (left side) and oris (right side) after full insertion in all subjects and the radiologically measured electrode facial nerve distances. The red line indicates the linear regression of the respective results, the gray shaded area around the regression line indicates the 0.95 confidence interval. R is the Spearman's correlation coefficient and p is the significance level of the correlation.

The scatter plots in Figure 9 show the relationship between the postoperatively determined EFND and the normalized amplitudes of the EMG responses of the orbicularis oculi (left side) and oris (right side) muscles measured for each electrode contact of all subjects after full insertion. The linear regression function of the data (red solid line) and the corresponding confidence interval (95%) show a clear decrease in normalized EMG responses with increasing EFND in both muscle groups. The Spearman's rank correlation coefficient R revealed a negative moderate correlation for the periocular EMG channel (R(106) = − 0.39, p < 0.001) and a negative strong correlation for the perioral EMG channel (R(106) =  − 0.6, p < 0.001). Both correlations were statistically significant.

## Discussion

In the present study, it was investigated whether the FNS evoked by electrical stimulation via the CI electrode can be used as a marker of the intracochlear position of the electrode array during and after insertion. It was hypothesized that electrode contacts with shorter distance to the facial nerve (EFND) can stimulate the nerve more effectively than contacts further away. The first part of this study included recordings of EMG responses during stepwise insertion of CI electrode arrays. The step size was two contacts at a time. After each step, stimulation pulses were emitted from the most apical contact on the array and the evoked EMG responses of the orbicularis oculi and oris muscles were recorded. In the second part of the study, stimulation pulses were sequentially emitted from each contact on the fully inserted electrode array and the evoked EMG responses were recorded. EFNDs of each patient were measured in postoperative fpVCT_SECO_ scans and compared with the intraoperatively recorded EMG responses.

The measurements of the EFND in the “cochlear view” of fpVCT_SECO_ scans showed sufficient feasibility. Interestingly, two types of slopes could be distinguished based on their minima, that is, the shortest EFND (Figure 4). One slope type showed the shortest EFND at contact 3 or 4, and only included the shortest electrode type (FLEX24). This electrode type was implanted in cases, in which the residual hearing should be preserved. Accordingly, the electrode should only partially cover the cochlear length to replace damaged inner hair cells in the range of the high frequencies, while keeping more apical hair cells functional. Therefore, the contacts closer to the apical end of the electrode were nearest to the facial nerve.

The other type of slope showed the shortest EFNDs at contact 6 and included all electrode types used for a full cochlear coverage (FLEX28, FLEXsoft, and Standard). The fact that the shortest EFND was measured at contact 6 for each of the three electrode types despite the difference in length between FLEX28 and FLEXsoft or Standard (see Table 2) can be explained by the fact that FLEXsoft and Standard were implanted in cochleae with longer CDL and both types have longer distances between contacts than the FLEX28.

In addition, the difference in length from the basal contact to contact 6 between the 28 mm and the 31.5 mm electrodes is relatively short (1.5 mm (5 × 0.3 mm)) and smaller than the distance between the contacts (2.1 or 2.4 mm). Consequently, the difference might not be large enough, that another contact other than number 6 will be closer to the facial nerve and though has a lower EFND. This knowledge might be used for further development and research. If further measurements of EFNDs confirm the present result, that electrode 6 has always the lowest EFND in cases of full cochlear coverage by long electrode types this can possibly be used to check correct insertion of the respected array in clinical routine by only measuring the EMG signal after insertion. In cases with partial cochlear coverage (e.g., for electro-acoustic stimulation) it might be possible to preoperatively determine, which contact should have the smallest EFND and though this contact should than be implanted closest to the facial nerve with consideration of the EMG signal.

In 8 of 9 patients, an electrically evoked EMG signal was successfully monitored during CI array insertion. As the insertion depth progressed, the distance between the most apical contact and the facial nerve initially decreased and the stimulation efficacy increased. This could be observed by the rise in EMG amplitudes as function of the number of inserted contacts. Thereafter, as insertion depth progressed, the distance between the electrode and the facial nerve increased again, which was reflected in the decrease of the EMG amplitudes. After the last insertion step, 4 of the subjects showed a new increase in EMG amplitudes. This occurred exclusively in subjects implanted with longer electrode types (Standard, FLEX28) and not in the 2 patients provided with a short FLEX24. Therefore, it can be assumed that this increase was due to a second approximation of the most apical contact with the facial nerve in the second cochlear turn. In 5 out of 8 subjects, there was concordance between the estimated number of inserted contacts, which showed an EMG peak above the threshold of 75% normalized EMG amplitude and the radiologically measured EFNDs. In one subject, whose shortest EFND was measured for an odd contact number, the EMG peak appeared at one of the even neighboring contacts. These results support the hypothesis that during increasing insertion passing of the electrode tip at the area of the facial nerve can be measured electromyographically.

ECochG is another electrophysiological measurement that can be used for real-time monitoring during cochlear implantation^14^. Although, ECochG is usually used to monitor cochlear trauma caused by CI electrode insertion, studies in humans and animals reported of increasing signal amplitudes with increasing electrode insertion depths, which were assumed to reflect the decreasing distance to areas with residual hearing function in the cochlear apex^15^. The advantage of the electromyographic measurement presented in this study over ECochG as measure for insertion depth is that the facial nerve can be used as an anatomical landmark to estimate the relative location of the electrode.

After full insertion, stimulation was performed from each electrode contact and the evoked EMG responses were recorded. The EMG profiles were normalized for better comparability between each subject and the two EMG channels used. To identify electrode contacts, which exhibited EMG peaks along the array an arbitrarily chosen threshold of 75% normalized EMG amplitude was used. The electrode contacts thus identified were compared with the electrode contacts that showed the shortest EFNDs in the postoperative fpVCT_SECO_ images.

Interestingly, the post insertion measurement showed multiple peaks of the EMG amplitude for some subjects (e.g., S7). This effect somewhat conflicts with the hypothesis and indicates that there may be more factors influencing FNS than EFND alone. Identification of these factors with the existing data is beyond the scope of this study. Theoretically, such peaks could indicate that the affected electrode contact has a lower contact resistance to tissue (i.e., impedance) than the electrode contacts with lower EMG amplitude but relatively low EFND. However, as the routine measurements of electrode impedances performed after implantation did not register higher values at these contacts, electrode impedance can be ruled out as a possible factor. Contrarily, these peaks may rather indicate paths of particularly high electrical conductivity between the respective electrode contact and the facial nerve. Hence, multiple peaks could be the result of an inconsistent distribution of electrically conductive media (e.g., perilymph) at the individual electrode contacts. Calloway et al.^15^ suggested that the increase in amplitudes in their ECochG measurements as the insertion depth of the electrode progressed may have also been due to a more favorable geometry with respect to the cochlear generators. Peaks or dips in the post insertion EMG amplitudes of the present study could thus also be a result of a favorable or unfavorable geometry between the individual electrode contacts and the facial nerve. However, according to the hypothesis, both EMG channels showed agreement between the shortest EFND and EMG peaks in 5 of 9 subjects (periocular: S1, S2, S5, S6, S8; perioral: S1, S2, S5, S7, S8). Besides the peaks that exceeded the threshold, the results of the post insertion measurements showed higher EMG responses for contacts lying on the more apical half of the electrode array. As observed in the data from the insertion measurements, this may reflect the decreasing EFND of those contacts, which are closer to the facial nerve within the second cochlear turn than the more basal contacts. This assumption is also supported by the statistically significant results of the correlation analysis, which showed moderate (periocular) and strong (perioral) negative correlation between the normalized EMG amplitudes and the EFNDs of each contact.

The comparison between the EMG response patterns post insertion with those during insertion shows that both procedures in some cases led to different response patterns in the same subjects (e.g., in S4). It should be noted that a direct comparison is only possible for three subjects (S3, S7 and S8), since for all other subjects both procedures were performed with different stimulation levels (see Table 3). Inaccuracies in the step size during the manually controlled stepwise insertion may also have led to biased response patterns that could not be reproduced post insertion.

The aim of this study was to prove the feasibility of the approach. The measurement presented is still under development and requires further improvements to increase precision. The following section is intended to provide starting points for future studies.

The “cochlear view” provided only EFNDs in a two-dimensional representation of the patient's anatomy. Close to the cochlea, however, the facial nerve exhibits a complex three-dimensional course. In addition, the CI array changes its position in three dimensions as it passes through the cochlear turns. The EFND measurements proposed in the present study should be considered as an estimate of the actual distance. Future studies should therefore investigate whether the EFND can be determined more precisely by considering the three-dimensional anatomy. Recent studies reported that patients with postoperative FNS had significantly lower distance and bone density between the upper basal turn of the cochlea and the labyrinthine segment of the facial nerve^33,34^. Therefore, besides the EFND future studies should also include parameters such as bone density, which can influence the propagation of the electric current. The stepwise insertion in the present study was performed manually. Thus, the number of contacts already inserted could only be estimated. In addition, an anatomically defined reference was missing for a precise indication of the length of the already inserted electrode array. The threshold 75% normalized EMG amplitude was chosen arbitrarily and is only a rough method to detect peaks. Modern methods of peak detection could achieve a better differentiation of different peaks (contact in close proximity to facial nerve, most apical contact in second cochlear turn). Future studies should consider changes to the stimulation paradigm that allow a higher number of repetitions which cause less fatigue to the facial nerve and the muscles innervated by it. This should improve the signal-to-noise ratio and additionally reduce the influence of outliers (electrical artifacts from the operating room, spontaneous movements of the patient) on peak detection. The used higher stimulation amplitudes resulted in broader electric fields in the cochlea and its surrounding anatomical structures, which might have influenced the accuracy of the system. Measurements in the orbicularis oris muscle yielded EMG responses with higher amplitudes, which resulted in a better signal-to-noise ratio. Therefore, compared with measurements in the orbicularis oculi muscle, lower current levels can be applied to stimulation, which should result in better spatial resolution of the EMG. Therefore, the authors suggest future studies to focus on EMG measurements in the orbicularis oris muscle.

## Conclusion

To the best of our knowledge, this is the first study to demonstrate the potential of using intentional FNS for real-time monitoring of the insertion status during and after cochlear implantation. This system might be helpful to the field of cochlear implantation, since by applying FNS stimulation, the position of the electrode can be determined quite precisely for at least the first six implanted contacts in the basal turn, which equals about 200 degrees of the cochlea. Because there is evidence that the EMG signal rises again as the stimulating contact approaches the facial nerve in the mid turn at about 540 degrees, this method could hypothetically detect an electrode tip-fold over as well. The system can easily be used to perform further measurements after surgery and during all wearing-time of the implant. After wound closure, the system can control, if manipulations during suturing of the periosteum, the subcutaneous tissue, and the skin might have led to an electrode displacement. In addition, if there is a suspicion for electrode extrusion after surgery, a measurement of the evoked EMG can be performed again. If the electrode has not been displaced, the highest EMG responses should be measured at the same stimulating contact as before.

On the long term, the system might be used in a radiation free cochlear implantation. Omitting radiation is of high benefit, especially for children^41^, since there is growing evidence from epidemiological studies that radiation exposure of the brain poses further substantial risks^42–44^. In this concept preoperative planning of the implantation, like measuring the CDL^45,46^, the examination of the course of facial nerve^47^ and the size of the temporal bone^48^, will be performed in MRI imaging. For intra- and postoperative control the presented system, may be used in combination with ECochG^49^ or electrically evoked compound action potentials^18^. If required, after the waiting time defined by the manufacturer, an MRI scan could be performed with special protocols to investigate the electrode position^21^.

## Abbreviations

CDL: Cochlear duct length
CI: Cochlear implant
ECochG: Electrocochleography
EFND: Electrode facial nerve distance
EMG: Electromyography
FNM: Facial nerve monitoring
FNS: Facial nerve stimulation
fpVCT: Flat-panel volume computer tomography
MRI: Magnet resonance imaging
RIB2: Research interface box 2
